# Supplementary material for: Sentiment correlation in financial news networks and associated market movements
Source: Sci Rep. 2021 Feb 4;11:3062. doi: 10.1038/s41598-021-82338-6 (PMC7862280; doi:10.1038/s41598-021-82338-6)
Supplement: Supplementary file 1 — Supplementary Information. [file 41598_2021_82338_MOESM1_ESM.pdf]

Supplementary information for *Sentiment Correlation in Financial News Networks and Associated Market Movements*

Authors: Xingchen Wan[1+*], Jie Yang[2,3+*], Slavi Marinov[4], Jan-Peter Calliess[1], Stefan Zohren[1], and Xiaowen Dong[1]
[1]Oxford-Man Institute of Quantitative Finance, University of Oxford, UK
[2]School of Public Health, Zhejiang University, China
[3]Harvard Medical School, Harvard University, USA
[4]Man AHL, UK
*xwan@robots.ox.ac.uk, jieynlp@gmail.com
+these authors contributed equally to this work.

Table S1. Company Abbreviation Map (in alphabetical order within each sector).

Sector	Abbreviation	Company
Financials (25)	AXP	American Express.
	AIG	American International Group.
	BAC	Bank of America.
	BMO	Bank of Montreal.
	BK	Bank of New York Mellon.
	BCS	Barclays.
	BRKA	Berkshire Hathaway.
	BX	Blackstone.
	BNPP	BNP.
	CS	Credit Suisse.
	DBKGN	Deutsche Bank.
	FMCC	Federal Home Loan Mortgage Corp.
	GS	Goldman Sachs.
	HSBA	HSBC.
	ING	ING Groep.
	JPM	JP Morgan.
	KKR	KKR.
	LEHMQ	Lehman Brothers.
	MA	MasterCard.
	MS	Morgan Stanley.
	RY	Royal Bank of Canada.
	RBS	Royal Bank of Scotland.
	SOCGEN	Societe Generale.
	UBSN	UBS.
	WFC	Wells Fargo.
Technology (16)	AAPL	Apple.
	BBRY	BlackBerry.
	CEOS	CeCors.
	CSCO	Cisco.
	ERICB	Ericsson.
	GARTNER	Gartner.
	GOOG	Google.
	HPQ	Hewlett Packard.
	IBM	IBM.
	INTC	Intel.
	MSFT	Microsoft.
	MCO	Moody 's Inc.
	NOK	Nokia.
	SSNLF	Samsung.
	SNE	Sony.
	YHOO	Yahoo.

Table S1 – continued from previous page

Sector	Abbreviation	Company
Materials (4)	AA	Alcoa.
	BLT	BHP.
	ISM	Inspiration Mining.
	RIO	Rio Tinto.
Communications (2)	VZ	Verizon Communications.
	VOD	Vodafone.
Healthcare (2)	JNJ	Johnson & Johnson.
	PFE	Pfizer.
Consumer Staples (6)	KO	Coca-Cola.
	COST	Costco Wholesale.
	PEP	Pepsi.
	PG	Procter & Gamble.
	TGT	Target Corp.
	WMT	Wal-Mart.
Consumer Discretionary (20)	AMZN	Amazon.
	BBY	Best Buy.
	BMWG	BMW.
	DAIGN	Daimler AG.
	DAL	Delta Airlines.
	EBAY	Ebay.
	FIA	Fiat Chrysler.
	GPS	Gap Inc.
	GM	General Motors.
	HD	Home Depot.
	HMC	Honda Motor.
	JCP	JC Penney.
	MCD	McDonald.
	NISSAN	Nissan.
	RENA	Renault.
	SBUX	Starbucks.
	TM	Toyota Motor.
	TVS	TVS Motor.
	UAL	United Continental Holdings.
	VOWG	Volkswagen AG.
Energy (5)	BP	BP.
	CVX	Chevron.
	RDSA	Royal Dutch Shell.
	EC	Ecopetrol.
	XOM	Exxon Mobil.
Industrials (7)	EAD	Airbus SE.
	BA	Boeing.
	CAT	Caterpillar.
	FDX	FedEx.
	GE	General Electric.
	UPS	United Parcel Service.
	UTX	United Technologies.

Table S2. Groups of companies based on the community detection on the news co-occurrence network of the first year ((in alphabetical order within each sector).

Group Number	Members	#Members
1	BAC, BCS, BK, BNPP, BX, CS, DBKGN, FMCC, GS, HSBA, ING, JPM, KKR, LEHMQ, MCO, MS, RBS, RY, SOCGEN, UBSN, UTX, WFC	22
2	BMWG, DAIGN, FIA, GM, HMC, NISSAN, RENA, TM, VOWG	9
3	AAPL, AMZN, BBY, CSCO, EBAY, EC, ERICB, GARTNER, GE, GOOG, HPQ, IBM, INTC, MA, MSFT, NOK, SSNLF, SNE, TVS, YHOO	20
4	COST, GPS, HD, JCP, TGT, WMT	6
5	AIG, BA, DAL, EAD, FDX, UAL, UPS	7
6	AXP, BBRY, BP, BRKA, CAT, CEOS, CVX, ISM, JNJ, KO, MCD, PEP, PFE, PG, RDSA, SBUX, XOM	17
7	AA, BLT, BMO, RIO, VOD, VZ	6

Table S3. Company pairs **not** belonging to the same ground-truth sector with edge weights above 75th percentile + 1.5 IQR in the out-sector weight distribution (Figure S1). Top 50 shown in descending magnitudes of edge weight.

Index	Company 1	Sector 1	Company 2	Sector 2
1	AAPL	Technology	AMZN	Consumer Discretionary
2	GPS	Consumer Discretionary	TGT	Consumer Staples
3	AA	Materials	DAL	Consumer Discretionary
4	DAIGN	Consumer Discretionary	EAD	Industrials
5	AA	Materials	UAL	Consumer Discretionary
6	AMZN	Consumer Discretionary	GOOG	Technology
7	AMZN	Consumer Discretionary	WMT	Consumer Staples
8	JCP	Consumer Discretionary	TGT	Consumer Staples
9	BRKA	Financials	KO	Consumer Staples
10	BBY	Consumer Discretionary	WMT	Consumer Staples
11	COST	Consumer Staples	GPS	Consumer Discretionary
12	AMZN	Consumer Discretionary	TGT	Consumer Staples
13	BRKA	Financials	PG	Consumer Staples
14	BA	Industrials	UAL	Consumer Discretionary
15	GPS	Consumer Discretionary	WMT	Consumer Staples
16	GE	Industrials	GM	Consumer Discretionary
17	AA	Materials	BA	Industrials
18	BA	Industrials	RY	Financials
19	EC	Energy	ING	Financials
20	EC	Energy	MSFT	Technology
21	AAPL	Technology	VZ	Communications
22	BBY	Consumer Discretionary	TGT	Consumer Staples
23	HD	Consumer Discretionary	TGT	Consumer Staples
24	GOOG	Technology	VZ	Communications
25	EBAY	Consumer Discretionary	MA	Financials
26	BRKA	Financials	MCO	Technology
27	MCO	Technology	SOCGEN	Financials
28	CSCO	Technology	RY	Financials
29	BNPP	Financials	MCO	Technology
30	KO	Consumer Staples	MCD	Consumer Discretionary
31	JCP	Consumer Discretionary	WMT	Consumer Staples
32	BA	Industrials	DAL	Consumer Discretionary
33	AMZN	Consumer Discretionary	MSFT	Technology
34	HD	Consumer Discretionary	WMT	Consumer Staples
35	EBAY	Consumer Discretionary	GOOG	Technology
36	AA	Materials	BMO	Financials
37	MCD	Consumer Discretionary	RY	Financials
38	EC	Energy	INTC	Technology
39	CEOS	Technology	KO	Consumer Staples
40	COST	Consumer Staples	JCP	Consumer Discretionary
41	MCD	Consumer Discretionary	PG	Consumer Staples
42	AMZN	Consumer Discretionary	SSNLF	Technology
43	ISM	Materials	RY	Financials
44	AMZN	Consumer Discretionary	GARTNER	Technology
45	BRKA	Financials	GE	Industrials
46	FIA	Consumer Discretionary	GE	Industrials
47	CAT	Industrials	MCD	Consumer Discretionary
48	BBRY	Technology	RY	Financials
49	CAT	Industrials	KO	Consumer Staples
50	BX	Financials	YHOO	Technology

Table S4. Company pairs belonging to the same ground-truth sector with edge weights above 75th percentile + 1.5 IQR in the in-sector weight distribution (Figure S1). Top 50 shown in descending magnitudes of edge weight.

Index	Company 1	Sector 1	Company 2	Sector 2
1	VOD	Communications	VZ	Communications
2	BLT	Materials	RIO	Materials
3	MSFT	Technology	YHOO	Technology
4	FDX	Industrials	UPS	Industrials
5	NISSAN	Consumer Discretionary	RENA	Consumer Discretionary
6	GS	Financials	MS	Financials
7	BMWG	Consumer Discretionary	DAIGN	Consumer Discretionary
8	KO	Consumer Staples	PEP	Consumer Staples
9	TGT	Consumer Staples	WMT	Consumer Staples
10	DAL	Consumer Discretionary	UAL	Consumer Discretionary
11	FIA	Consumer Discretionary	GM	Consumer Discretionary
12	GOOG	Technology	MSFT	Technology
13	BNPP	Financials	SOCGEN	Financials
14	GOOG	Technology	YHOO	Technology
15	BA	Industrials	EAD	Industrials
16	BX	Financials	KKR	Financials
17	BCS	Financials	RBS	Financials
18	BK	Financials	BX	Financials
19	SSNLF	Technology	SNE	Technology
20	AMZN	Consumer Discretionary	BBY	Consumer Discretionary
21	HMC	Consumer Discretionary	TM	Consumer Discretionary
22	AAPL	Technology	SSNLF	Technology
23	DAIGN	Consumer Discretionary	FIA	Consumer Discretionary
24	MCD	Consumer Discretionary	SBUX	Consumer Discretionary
25	DAIGN	Consumer Discretionary	RENA	Consumer Discretionary
26	CS	Financials	UBSN	Financials
27	AAPL	Technology	GOOG	Technology
28	COST	Consumer Staples	WMT	Consumer Staples
29	HMC	Consumer Discretionary	NISSAN	Consumer Discretionary
30	AMZN	Consumer Discretionary	EBAY	Consumer Discretionary
31	GARTNER	Technology	NOK	Technology
32	GS	Financials	JPM	Financials
33	AXP	Financials	MA	Financials
34	HPQ	Technology	IBM	Technology
35	RDSA	Energy	XOM	Energy
36	AAPL	Technology	GARTNER	Technology
37	CVX	Energy	XOM	Energy
38	NISSAN	Consumer Discretionary	TM	Consumer Discretionary
39	BMWG	Consumer Discretionary	VOWG_P	Consumer Discretionary
40	DAIGN	Consumer Discretionary	VOWG_P	Consumer Discretionary
41	BAC	Financials	WFC	Financials
42	GARTNER	Technology	MSFT	Technology
43	NOK	Technology	SSNLF	Technology
44	BCS	Financials	LEHMQ	Financials
45	MSFT	Technology	NOK	Technology
46	BAC	Financials	GS	Financials
47	COST	Consumer Staples	TGT	Consumer Staples
48	CS	Financials	GS	Financials
49	AAPL	Technology	NOK	Technology
50	BAC	Financials	JPM	Financials

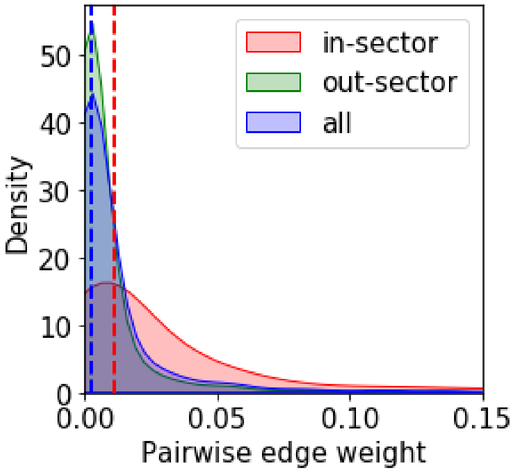

Figure S1. Probability distributions of pairwise edge weights between companies in the co-occurrence networks of companies belonging to the same sector (in-sector), companies belonging to the different sectors (out-sector) and overall distributions (all). The dotted vertical lines denote the population medians for each distribution. Note that the *out-sector* and *all* medians overlap because most edges are out-sector, since it is more likely for two arbitrary companies to belong to different sectors than the same sector.

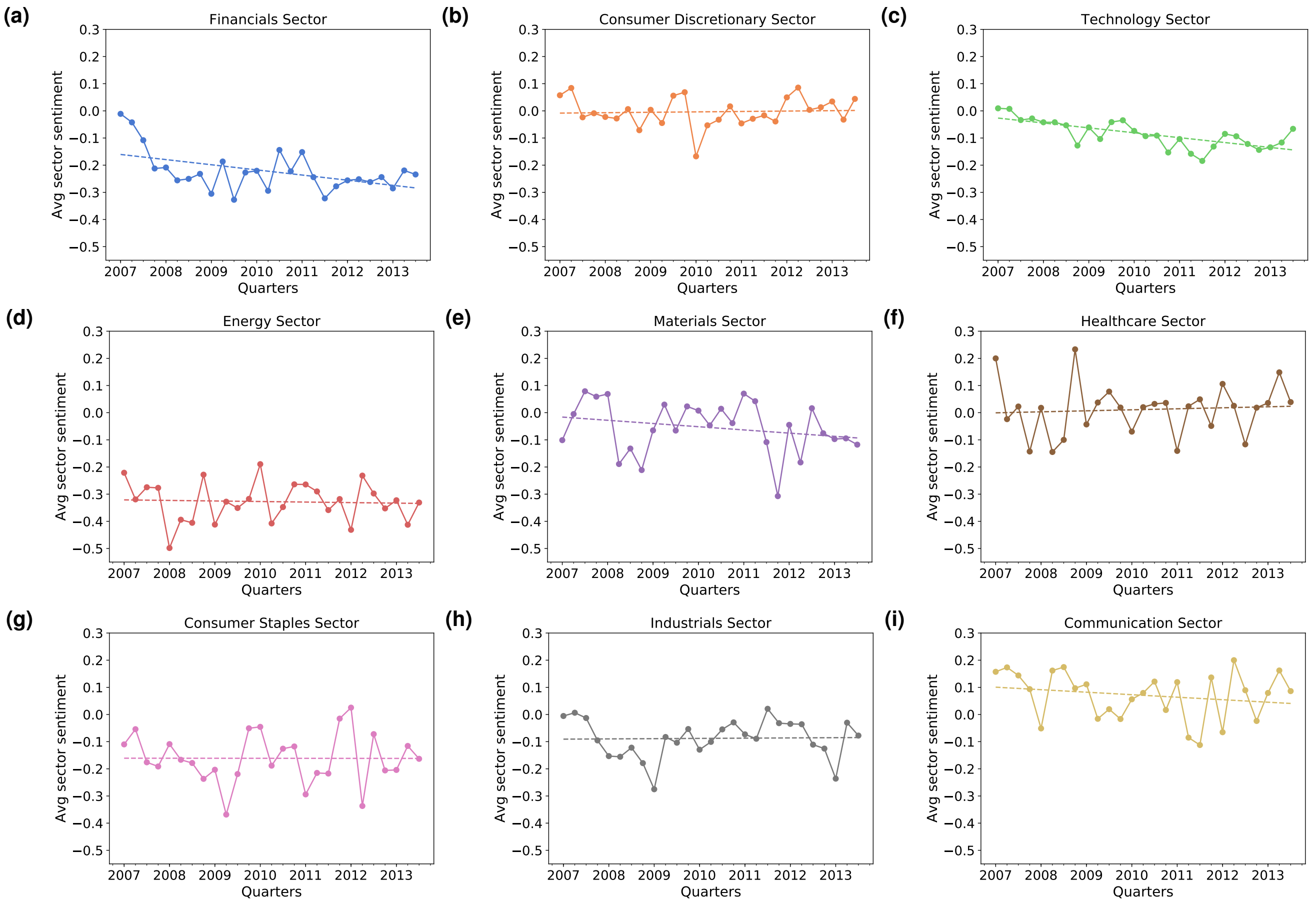

Figure S2. Evolution of average sentiment for the 9 sectors.

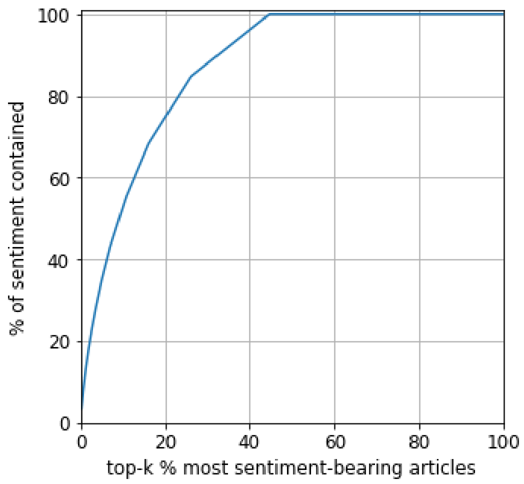

Figure S3. Fraction of total non-neutral sentiment (i.e. either positive or negative sentiment) towards the 87 target companies versus the fraction of non-neutral sentiment-bearing articles. It is clear that the a small number of articles account for a disproportionately large amount of sentiment directed to the target companies, with top 9.2% articles accounting for 50% sentiments and top 32.6% articles accounting for 90% sentiments. Slightly more than half of the articles do not contain non-neutral sentiment to the target companies.

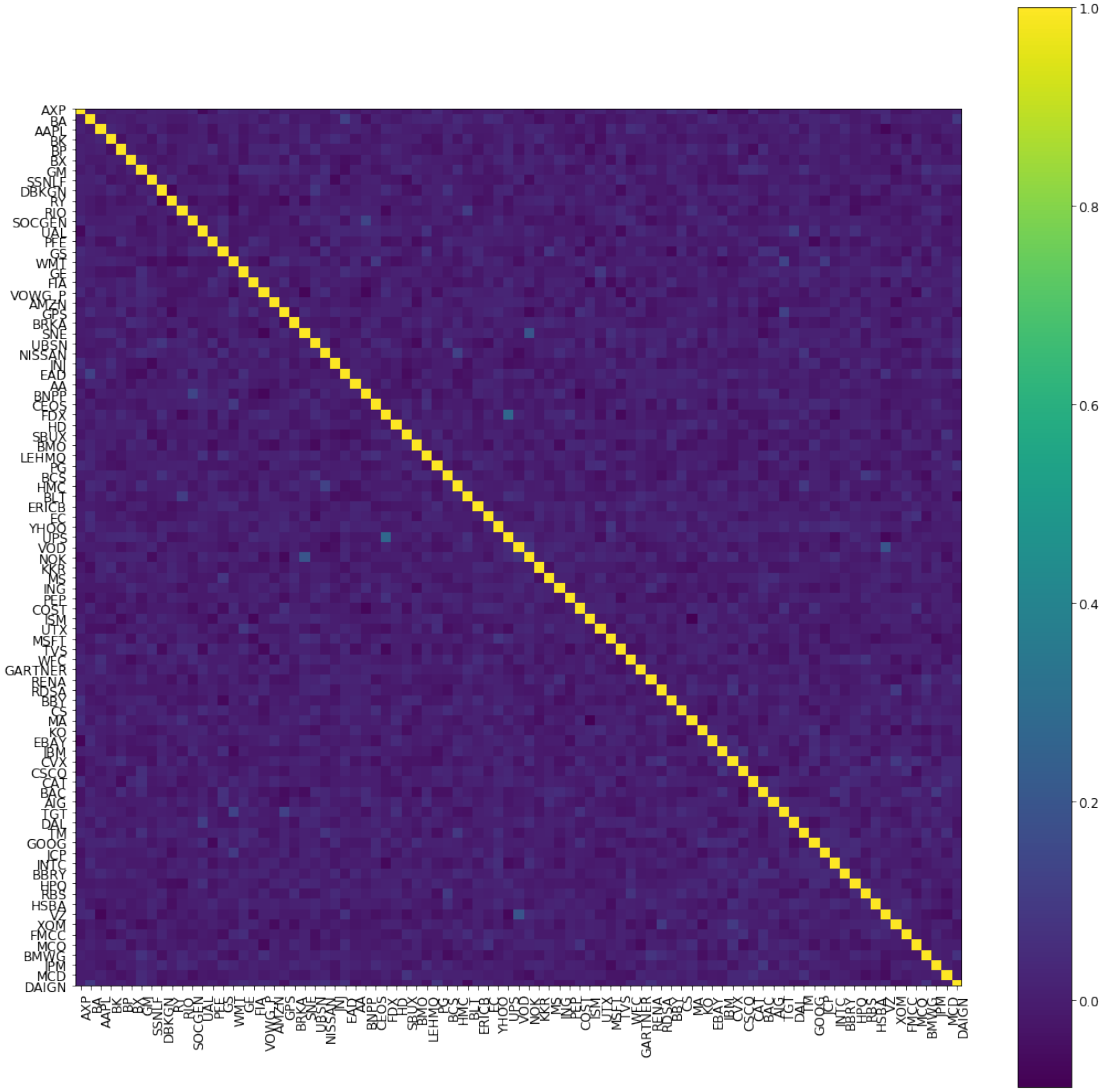

Figure S4. Pair-wise correlation of the sentiment event time series of all the 87 target companies

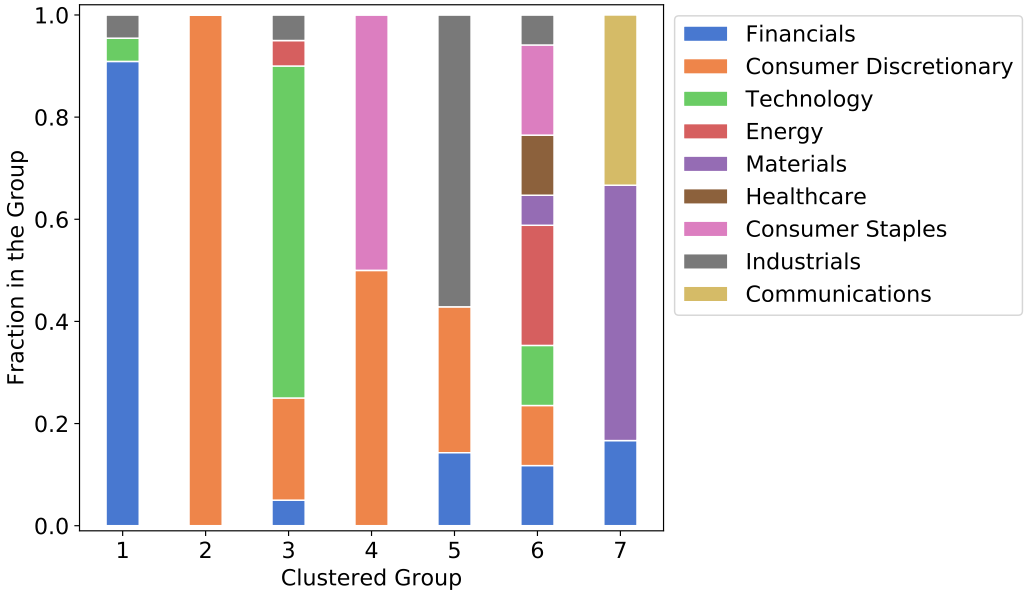

Figure S5. Distribution of sectors in 7 clustered groups in Figure 1(c) in the main text.

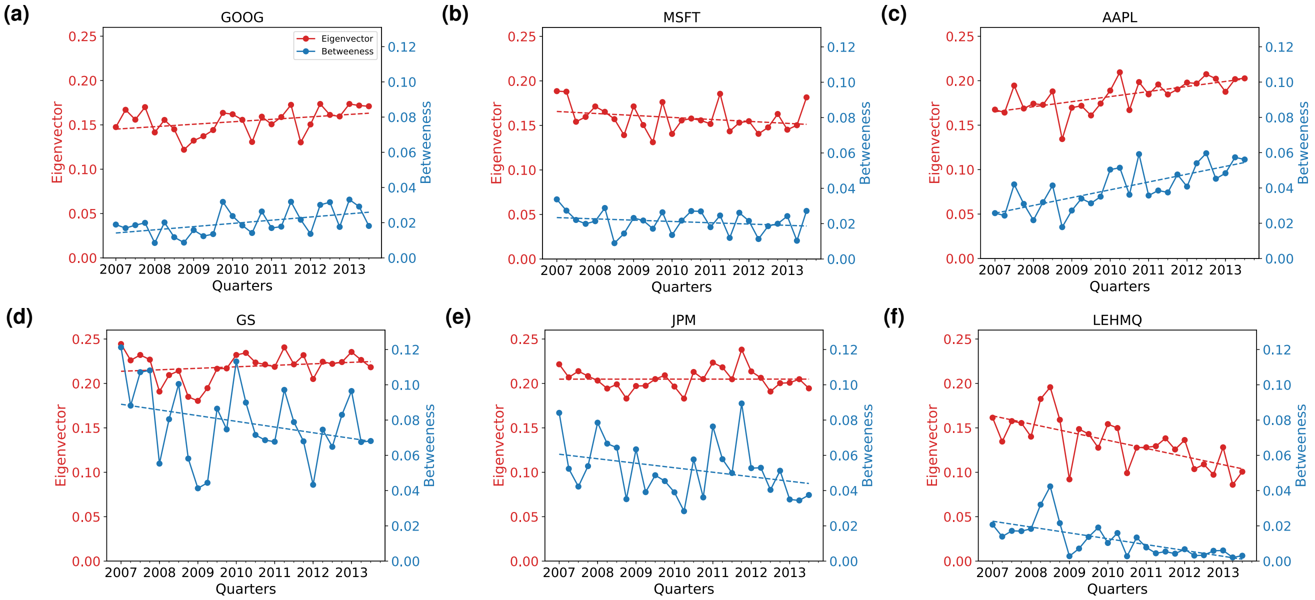

Figure S6. Evolution of the eigenvector (red) and betweenness (blue) centrality measures of several representative companies from the "Technology" and "Financial Services" sectors: (a) Google; (b) Microsoft; (c) Apple; (d) Goldman Sachs; (e) JP Morgan; (f) Lehman Brothers.

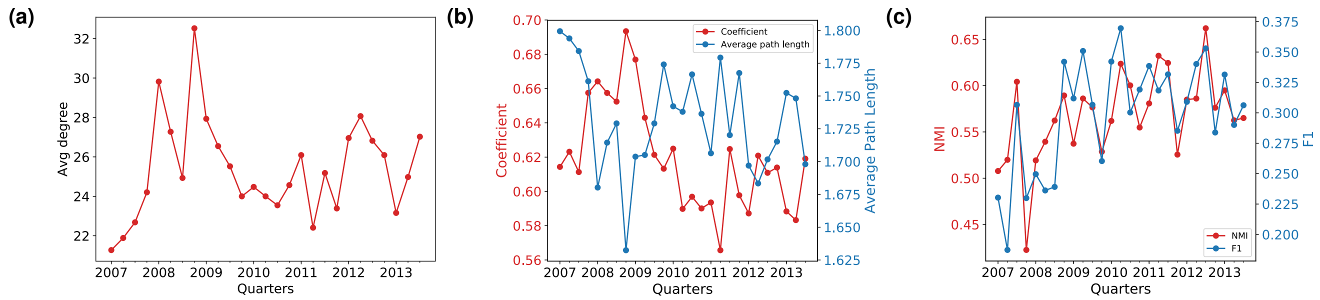

Figure S7. Evolution of news co-occurrence network features and dynamic comparison with ground-truth sectors. (a) Average degree; (b) Clustering coefficient and average path length; (c) Auto-detected groups vs. ground-truth sectors.

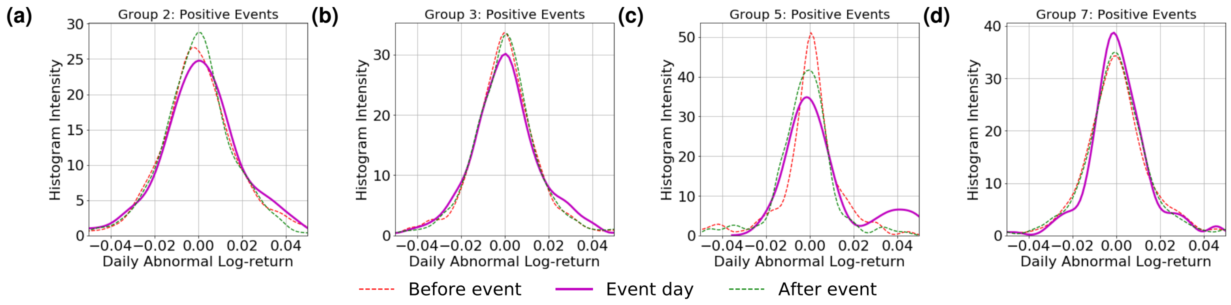

Figure S8. Probability density function of **AR** of companies in several groups, computed over all group-aggregated **positive** sentiment events over the time period: (a) Group 2, (b) Group 3, (c) Group 5, (d) Group 7.

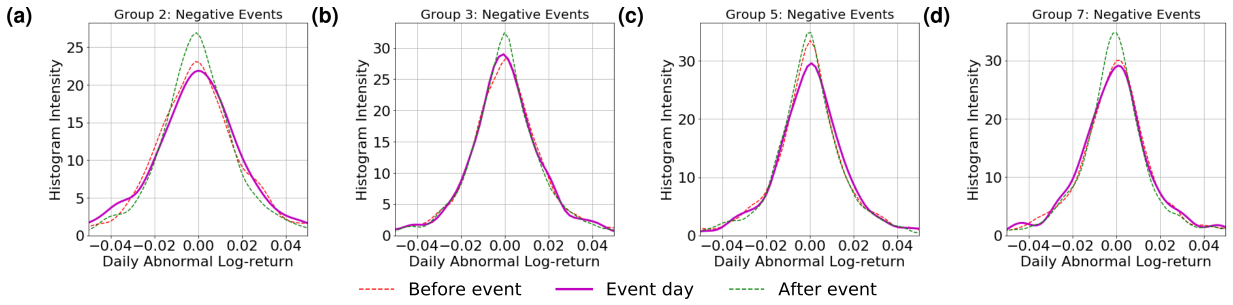

Figure S9. Probability density function of **AR** of companies in several groups, computed over all group-aggregated **negative** sentiment events over the time period: (a) Group 2, (b) Group 3, (c) Group 5, (d) Group 7.

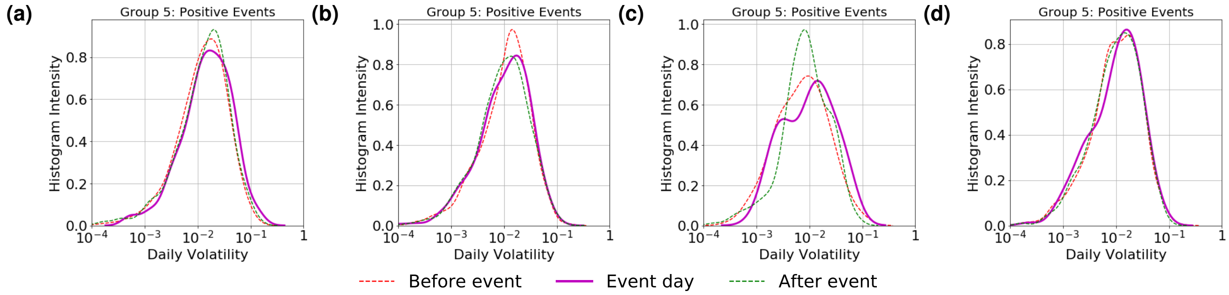

Figure S10. Probability density function of **Daily realised volatility** of companies in several groups, computed over all group-aggregated **positive** sentiment events over the time period: (a) Group 2, (b) Group 3, (c) Group 5, (d) Group 7.

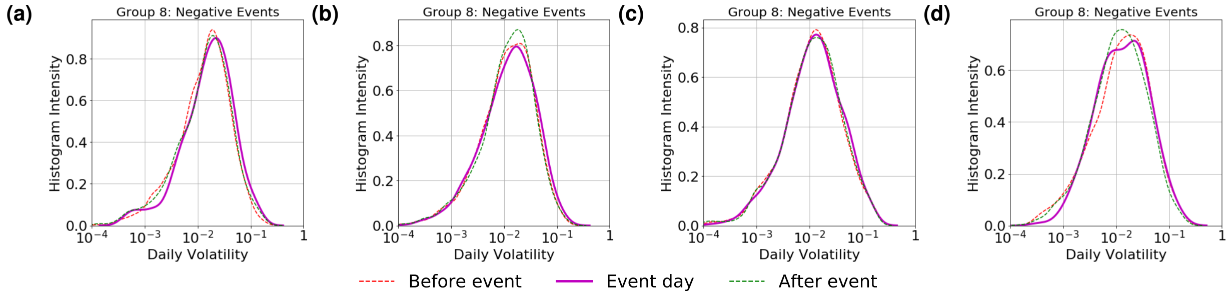

Figure S11. Probability density function of **Daily realised volatility** of companies in several groups, computed over all group-aggregated **negative** sentiment events over the time period: (a) Group 2, (b) Group 3, (c) Group 5, (d) Group 7.
